# Research on Alkali-Activated Slag Stabilization of Dredged Silt Based on a Response Surface Method

**DOI:** 10.3390/ma17174410

**Published:** 2024-09-06

**Authors:** Qizhi Hu, Wei Yao, Gaoliang Tao

**Affiliations:** 1 School of Civil Architecture and Environment, Hubei University of Technology, Wuhan 430068, China; hqz0716@163.com (Q.H.); tgl1979@126.com (G.T.); 2 Hubei Bridge Safety Monitoring Technology and Equipment Technology Engineering Research Center, Wuhan 430068, China

**Keywords:** dredged silt, blast furnace slag, alkali activation, response surface method, microscopic characteristics

## Abstract

To improve the resource utilization of dredged silt and industrial waste, this study explores the efficacy of using ground granulated blast furnace slag (GGBS), active calcium oxide (CaO), and sodium silicate (Na_2_O·nSiO_2_) as alkali activators for silt stabilization. Through a combination of addition tests, response surface method experiments, and microscopic analyses, we identified key factors influencing the unconfined compressive strength (UCS) of stabilized silt, optimized material ratios, and elucidated stabilization mechanisms. The results revealed the following: (1) CaO exhibited the most pronounced stabilization effect, succeeded by Na_2_O·nSiO_2_, whereas GGBS alone displayed marginal efficacy. CaO-stabilized silt demonstrated rapid strength augmentation within the initial 7 d, while Na_2_O·nSiO_2_-stabilized silt demonstrated a more gradual strength enhancement over time, attributable to the delayed hydration of GGBS in non-alkaline conditions, with strength increments noticeably during later curing phases. (2) Response surface analysis demonstrated substantial interactions among GGBS-CaO and GGBS-Na_2_O·nSiO_2_, with the optimal dosages identified as 11.5% for GGBS, 4.1% for CaO, and 5.9% for Na_2_O·nSiO_2_. (3) X-ray diffraction (XRD) and scanning electron microscopy (SEM) analyses clarified that the hydration reactions within the GGBS-Na_2_O·nSiO_2_ composite cementitious system synergistically enhanced one another, with hydration products wrapping, filling, and binding the silt particles, thereby rendering the microstructure denser and more stable. Based on these experimental outcomes, we propose a microstructural mechanism model for the stabilization of dredged silt employing GGBS-CaO-Na_2_O·nSiO_2_.

## 1. Introduction

With the expansion of large-scale urban construction projects along China’s coastline, there has emerged an urgent need to manage the vast quantities of dredged silt generated [1,2]. Characterized by high water content, extensive porosity, significant compressibility, and low bearing capacity, and laden with considerable amounts of organic matter and pollutants [3,4,5], the accumulation of dredged silt not only depletes urban land resources but also poses substantial environmental risks. Presently, prevalent methods for sludge treatment encompass natural processing, thermal treatment, electro-osmotic consolidation, and chemical stabilization. Among these, chemical stabilization, distinguished by its convenience, versatility in material selection, and cost-effectiveness [6,7], has emerged as the predominant technology for the recycling of dredged silt. This technique entails the addition of stabilizers that initiate a cascade of physicochemical reactions within the silt, thereby reducing its water content and significantly boosting its strength to fulfill the specifications required for use as a roadbed filler [8].

In the stabilization of dredged silt, ordinary Portland cement is frequently employed as a solidifying agent for soft soils [8,9]. However, cement production entails considerable carbon emissions and substantial energy consumption, with each ton of cement necessitating approximately 5000 MJ of energy and releasing 0.95 t of CO_2_, contributing from 5% to 8% of global greenhouse gas emissions [10,11]. Despite its mechanical robustness, cement-stabilized soil frequently exhibits poor durability, water instability, considerable shrinkage, and a propensity for cracking [12,13]. To bolster the high-quality economic advancement of coastal cities, an urgent exploration of sustainable and efficacious green alternatives to cement and lime for civil engineering applications is necessary [14,15]. Numerous scholars have investigated the formulation ratios of composite stabilizers, endeavoring to enhance the stabilization effects on silty soil by incorporating additional components into the cement mix. Blast furnace slag, a silicate byproduct of industrial iron smelting, possesses mineral components akin to those of cement clinker and displays potential hydraulic activity, activatable through alkali activation [16]. Investigations by Liu et al. [17] employing scanning electron microscopy to analyze the micro-morphology of granulated blast furnace slag in varied hydration environments revealed enhanced reactivity during room temperature alkali activation, culminating in more complete hydration of the slag particles. Yi et al. [18] discovered that the strength of activated blast furnace slag-stabilized soil could achieve 2.4 to 3.2 times that of cement-stabilized soil after 90 d. Liang and colleagues [19] employed blast furnace slag powder and cement as a composite stabilizer for zinc-contaminated silty soil, determining that a mixture of 15% cement and 10% slag provided optimal zinc fixation, alongside the greatest strength and stability of the stabilized soil. He Jun et al. [20] used alkali slag and blast furnace slag for silt stabilization, attaining an unconfined compressive strength of 1228.3 kPa after seven days with 30% alkali slag and 8% slag. In evaluating and selecting additives, orthogonal experimental methods are frequently employed, which economize on time and mitigate the experimental workload to some extent but do not outline clear functional relationships between additive dosages and response values across the entire region, thereby precluding the determination of optimal ratios for achieving maximum response values. In contrast, response surface methodology amalgamates mathematical and statistical insights, facilitating the design of experiments, the establishment of fitting models, and the assessment of interactions between variables [21]. Moreover, the precision of the Box–Behnken design in response surface methodology has gained widespread recognition in domains such as concrete production, cost analysis, and pharmaceutical testing [22].

By employing response surface methodology, this study synergistically integrated mathematical and statistical approaches to investigate the stabilization of dredged silt using alkaline-activated slag. GGBS-CaO-Na_2_O·nSiO_2_ was developed as a composite solidifier, supplanting traditional cement, grounded on outcomes from single-addition experiments. Optimal ratios of GGBS, CaO, and Na_2_O·nSiO_2_ were ascertained, and their effects on the unconfined compressive strength of the stabilized silt were evaluated. The mechanical properties and microstructural strength mechanisms were further explained through X-ray diffraction (XRD) and scanning electron microscopy (SEM). The employment of industrial waste in treating dredged silty soil not only conserves resources like cement and lime but also advances the resourceful utilization of industrial waste and dredged silt. This methodology embodies the principle of “treating waste with waste”, underpinning sustainable environmental practices.

## 2. Materials and Methods

### 2.1. Test Materials

**Figure 1:**
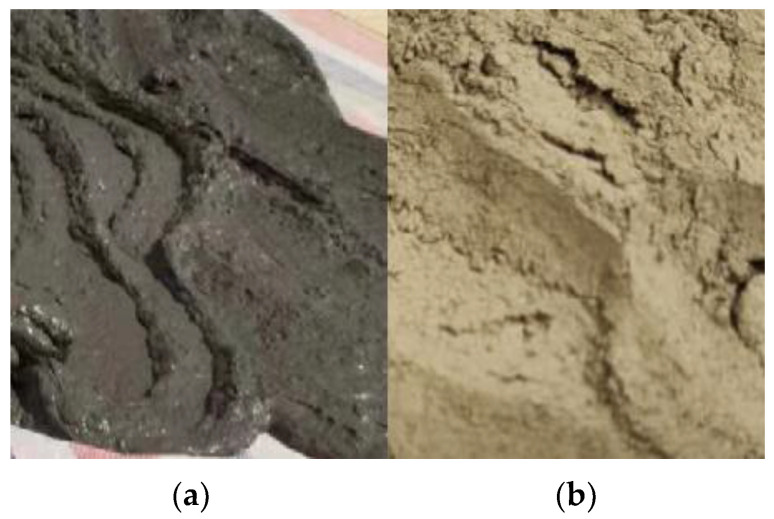
Dredged silt. (**a**) Undisturbed dredged silt and (**b**) dried dredged silt.

The dredged silt samples used in this experiment were sourced from the Xunsi River in Wuhan, China. This dredged silt exhibits a gray-black hue and is in a fluid-plastic state, as illustrated in Figure 1. Prior to initiating the experiment, the fundamental physical properties and principal chemical components of the soil samples were quantified, as detailed in Table 1 and Table 2. In Table 1, the liquid limit, defined as the moisture content at which soil transitions from a plastic to a liquid state, and the plastic limit, identified as the moisture content initiating plastic behavior in soil, are quantified. The plasticity index is derived by the subtraction of the plastic limit from the liquid limit. Meanwhile, the liquidity index, which quantifies the soil’s consistency relative to its liquid and plastic limits, is calculated by taking the soil’s natural moisture content, deducting the plastic limit, and dividing it by the plasticity index.

**Figure 2:**
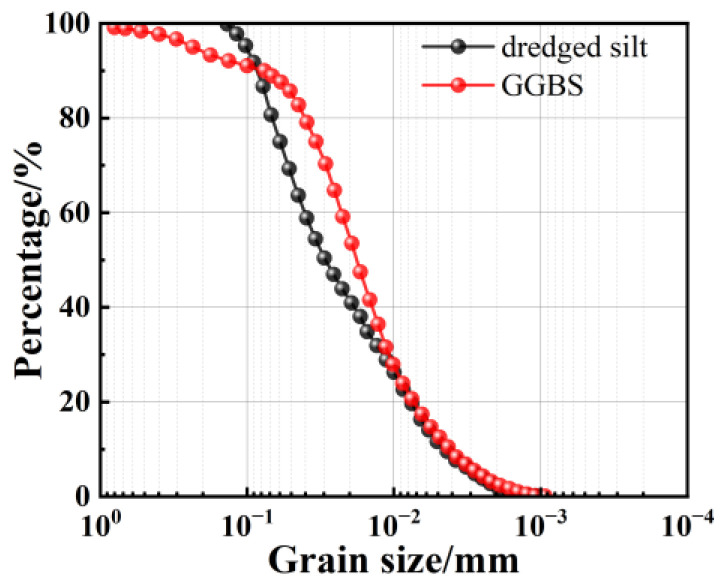
Particle grading curve of GGBS and dredged silt.

The S95 slag powder employed in the experiment was procured from the Jiyuan Steel Plant (Jiyuan, China). The particle size distribution curves for GGBS and dredged silt are depicted in Figure 2, while the primary chemical components are listed in Table 2. Silicate (Huasheng Chemical Reagent Co., Ltd., Tianjin, China) and calcium oxides (Sinopharm, Beijing, China) served as alkaline activators in the experiment, with all reagents being of analytical grade. Tap water was used for the experimental procedures.

### 2.2. Sample Preparation

**Figure 3:**
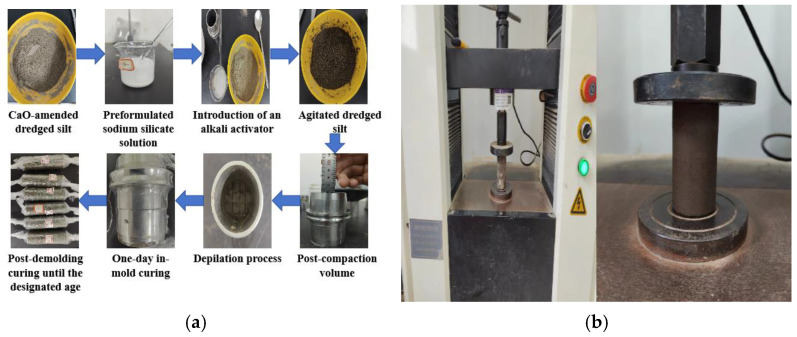
(**a**) Preparation of specimens, and (**b**) unconfined compressive strength testing.

In accordance with the standard for geotechnical testing methods (GB/T 50123-2019) [23], the ratio of the sample’s height (h) to its diameter (D) should lie within the range of 2.0 to 2.5, accommodating diameters of 39.1 mm, 61.8 mm, and 101.0 mm. For this particular test, with a diameter of 39.1 mm, the height is accordingly set at 80 mm. The sample preparation process encompasses the following steps: ① extract large impurities such as plastic, leaves, and branches from the dredged silt and air dry the silt to the predetermined moisture content; ② pulverize the air-dried silt and sift it through a 2 mm sieve; ③ oven dry the sieved silt at 105 °C for a duration exceeding 24 h; ④ homogenize the silt with the solidifying agent according to the specified test ratios, stir thoroughly, and then seal and allow to stand for 24 h; ⑤ employ the layered compaction method for sample preparation. Prior to molding, uniformly apply petroleum jelly to the interior of the mold. Compact the silt amalgamated with the solidifying agent into four distinct layers within the mold to form cylindrical samples measuring 39.1 mm in diameter and 80 mm in height, producing three parallel samples for each test group; ⑥ following preparation, encase the samples in cling film to mitigate moisture evaporation, position them in a standard curing chamber (temperature (20 ± 1) °C, humidity (98 ± 1)%), demold after 24 h, verify the samples’ integrity, reseal, and continue curing until the designated durations for unconfined compressive strength testing are reached. The experiment procedure is shown in Figure 3.

The unconfined compressive strength test (UCS) serves as a prevalent method for assessing the mechanical properties of various materials, including soil, concrete, and rock. This test primarily determines the maximum axial compressive strength that a material can withstand without lateral support. Typically, samples are cylindrical with smooth, flat ends to ensure a uniform stress distribution during loading. The prepared sample is positioned between the compression plates of the WDW-10E microcomputer-controlled electronic universal testing machine (Chenda Testing Machine Manufacturing Co., Ltd. (Jinan, China)), ensuring perfect alignment of the sample’s axis with the load application direction. The load is administered uniformly at a rate of 1 mm/min until the sample fails. The testing machine automatically documents the load and deformation experienced by the sample throughout the process, halting upon achieving 3% to 5% axial deformation subsequent to the peak stress. The peak axial stress, or in its absence, the axial stress at 20% axial strain, is designated as the unconfined compressive strength of the sample. The unconfined compressive strength of the specimen was calculated using Equation (1).

(1) $$q_{u}=\frac{P}{A}$$

In Equation (1), *q_u_* denotes the specimen’s unconfined compressive strength in MPa, *P* represents the maximum failure load in N, and *A* is the cross-sectional area in mm^2^.

### 2.3. Single-Addition Experiment

To determine the impact of each stabilization material on the unconfined compressive strength (UCS) of dredged silt, single-addition tests were performed by incorporating three different materials into the dredged silt individually, designated as GGBS-stabilized silt, CaO-stabilized silt, and Na_2_O·nSiO_2_-stabilized silt. After curing to the respective ages, tests for unconfined compressive strength were conducted. UCS was used as the criterion to evaluate the stabilization effects of the materials on the dredged silt and to ascertain the optimal dosage ranges for the stabilization materials. The details of the single-addition test procedure can be found in Table 3, where three replicate samples were taken for each test specimen.

### 2.4. RSM Experiment

The influence of alkali-activated slag on the macroscopic properties of stabilized dredged silt and its viability as a stabilizing agent are clarified in this study, which examines the effects of various factors on the early and prolonged strength of the stabilized soil. The proportions of GGBS (the ratio of GGBS mass to the dry mass of dredged silt), CaO (the ratio of CaO mass to the dry mass of dredged silt), and Na_2_O·nSiO_2_ (the ratio of Na_2_O·nSiO_2_ mass to the dry mass of dredged silt) are employed as independent variables in the experiment, designated as A, B, and C, respectively. The compressive strength of the stabilized soil at 7 and 28 d served as the response variable, denoted by Y.

### 2.5. X-ray Diffraction Test (XRD)

The instrument used for the experiment was a Bruker D8 ADVANCE X-ray diffractometer (Bruker, Billerica, MA, USA). The experiment used Cu Kα radiation with a wavelength of 1.5418 Å. The scanning parameters were set with an angle range of 10° to 80° and a speed of 10°/min. Samples were collected from untreated soil and the optimal mix proportions at intervals of 7 and 28 d. The samples were processed by oven-drying the fragments at a low temperature of 40 °C for 48 h. The dried samples were subsequently pulverized into powder using an agate mortar and sifted through a 0.075 mm sieve.

### 2.6. Scanning Electron Microscopy Test (SEM)

The instrument used for SEM was a ZEISS Sigma300 scanning electron microscope (ZEISS, Oberkochen, Germany). Samples were extracted from the damaged portions of the unconfined compressive strength tests and sectioned into small cubes of approximately 1 cm^3^. These cubes were positioned in an oven and dried for over 48 h to thoroughly eliminate the free water and bonded water from the samples. Prior to testing, the samples were fractured to yield clean and uniform fracture surfaces. Subsequently, these small soil fragments were gold-coated. The gold-coated samples were positioned in the SEM instrument, and following the establishment of a vacuum, scanning observations were undertaken.

## 3. Results

### 3.1. Results and Analysis of Single-Addition Experiments

**Figure 4:**
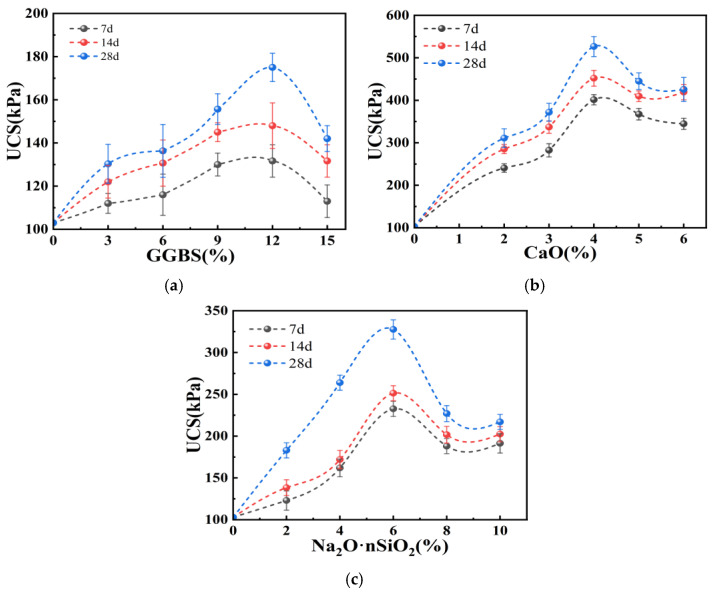
UCS of single-addition stabilized dredged silt: (**a**) GGBS, (**b**) CaO, and (**c**) Na_2_O·nSiO_2_.

Figure 4a depicts the influence of GGBS on the unconfined compressive strength of dredged silt. The graph depicts a trend where, as the GGBS content increases, the compressive strength of the stabilized silt initially rises and subsequently diminishes. Specifically, the strength of the stabilized silt gradually escalates with the GGBS content below 12%; however, beyond this threshold, the strength begins to wane. This trend is ascribed to the formation of cohesive hydration products, notably calcium silicate hydrate (CSH) and calcium aluminate hydrate (CAH), arising from the hydration of GGBS [24]. With increasing GGBS content, a greater quantity of these hydration products forms, thereby enhancing the compressive strength. At a constant GGBS content, the strength of the stabilized silt progressively increases with curing age. For example, at a 12% GGBS content, the compressive strength of the samples is 131.67 kPa at 7 d, escalates to 148 kPa at 14 d, and peaks at 175 kPa by 28 d, marking a 33% increase from the 7 d strength. However, at any given curing age, the maximum strength achieved with 12% GGBS content peaks at only 175 kPa, falling short of construction requirements [25]. Consequently, it is imperative to incorporate alkali activators into GGBS to enhance hydration [16]. According to experimental outcomes, the optimal GGBS content ranges from 9% to 15%.

Figure 4b portrays the impact of CaO on the unconfined compressive strength of dredged silt. As depicted in Figure 4b, with the incremental addition of CaO, the strength of the stabilized silt initially rises and subsequently declines, peaking at a CaO content of 4%, markedly exceeding the effect of GGBS-stabilized silt. At a CaO content of 4%, the strength of the stabilized silt significantly intensifies; however, beyond this threshold, further increases in CaO content result in a decline in strength. This phenomenon is attributable to the intense hydration reaction of CaO upon addition to soft soil, resulting in substantial production of Ca^2+^ ions. These ions promote the formation of calcium silicate hydrate (CSH) and calcium aluminate hydrate (CAH) [26]. However, as the hydration of CaO progresses, excessive precipitation of Ca(OH)_2_ crystals ensues, engendering voids within the soil structure and diminishing the stabilization efficacy of CaO on the silt. Consequently, based on experimental findings, the optimal CaO content ranges between 3% and 5%.

Figure 4c portrays the impact of Na_2_O·nSiO_2_ on the unconfined compressive strength of dredged silt. The graph depicts an initial increase followed by a decrease in the strength of the samples with escalating Na_2_O·nSiO_2_ content, peaking at 6%. Once the content exceeds 6%, the strength markedly declines, detrimentally impacting the stabilization of the dredged silt. This behavior is attributed to the robust adsorptive properties and the formation of cementitious substances during the hydration reaction of Na_2_O·nSiO_2_, which significantly contributes to silt stabilization [27]. The hydration reaction of Na_2_O·nSiO_2_ yields a substantial amount of fibrous material that permeates the stabilized soil, filling voids and augmenting the soil’s density and structural integrity. However, as the content increases, the diminished space for hydration reactions, resulting from the filled voids, impedes further Na_2_O·nSiO_2_ hydration. Consequently, based on experimental outcomes, the recommended Na_2_O·nSiO_2_ content range is 4% to 8%.

Based on the results of the single-addition experiments involving GGBS, CaO, and Na_2_O·nSiO_2_, it is clear that all three materials positively influence the stabilization of dredged silt. Among these, CaO exhibits the most pronounced solidifying effect, succeeded by Na_2_O·nSiO_2_, with GGBS demonstrating the least effectiveness. Taking into account the solidifying effects and economic considerations of the three materials, the optimal content ranges for the dredged silt in this study are identified as 9% to 15% for GGBS, 3% to 5% for CaO, and 4% to 8% for Na_2_O·nSiO_2_.

### 3.2. Results and Analysis of RSM Experiments

Employing the Box–Behnken design within Design-Expert software (Version: 13.0.5.0 64-bit), a three-factor, three-level experiment was executed to explain the impacts of each experimental factor and their synergistic interactions on the strength of the stabilized soil. The coding and level configuration of the independent variables are shown in Table 4.

The response surface methodology incorporates 17 experimental groups. Unconfined compressive strength tests on the stabilized dredged silt were performed at two distinct curing periods: 7 and 28 d. The response values, denoted as Y_7d_ and Y_28d_, are expressed in units of kilopascals (kPa). The experimental groups and their corresponding results are detailed in Table 5.

Using Design-Expert software, the experimental results presented in Table 5 were subjected to a second-order polynomial regression analysis. The second-order regression equation and the results of the variance analysis for each term are detailed in Table 6.

This study employed the F-distribution to evaluate the significance of the regression outcomes. During the analysis, it is imperative to first establish the significance level α. In Table 6, should the *p*-value fall below α, the corresponding experimental result is deemed significantly different. Conversely, if not significantly different, it can be excluded from the optimization analysis. In this study, the significance level α was established at 0.05. As shown in Table 6, the model F-values for Y_7d_ and Y_28d_ are 48.31 and 61.02, respectively, with *p*-values both under 0.0001, signifying that the regression models possess high significance. For Y_7d_, the significance ranking of the single factors is B > C > A, denoting CaO content > Na_2_O·nSiO_2_ content > GGBS content. The significance ranking of the factor interactions is AB > AC > BC, suggesting that the 7 d unconfined compressive strength of the stabilized soil predominantly relates to the CaO and GGBS contents. For Y_28d_, the significance ranking of the single factors is B > A > C, signifying CaO content > GGBS content > Na_2_O·nSiO_2_ content. The significance ranking of the factor interactions is AC > BC > AB, indicating that the 28 d unconfined compressive strength of the stabilized soil is chiefly associated with the GGBS and Na_2_O·nSiO_2_ contents. Because the Design-Expert software was employed, non-significant terms were systematically excluded to formulate the second-order polynomial regression equations between the GGBS content (A), CaO content (B), Na_2_O·nSiO_2_ content (C), and the unconfined compressive strength of stabilized soil at 7 and 28 d, as outlined in Equations (2) and (3).

Y_7d_ = 680.4 − 16.62A + 25.75B + 18.13C + 62AB − 10.75AC + 2BC − 76.08A^2^ − 89.33B^2^ − 93.57C^2^ (2)

Y_28d_ = 1022.4 − 34.25A + 40.75B − 21.75C − 62AB − 13.25AB − 47.25BC − 17.25BC − 102.08A^2^ − 114.58B^2^ − 97.07C^2^ (3)

Table 7 displays the outcomes of the model reliability analysis. The proximity of the model correlation coefficient (R^2^) and the adjusted determination coefficient (Adjusted R^2^) confirm the adequacy of the regression equation’s fit. Furthermore, a coefficient of variation (C.V.) below 10, a signal-to-noise ratio (Adequate Precision) exceeding 4, and a disparity less than 0.2 between the Adjusted R^2^ and the Predicted R^2^ emphasize the high accuracy and reliability of the experiments. As shown in Table 7, the R^2^ values for the models established for 7 and 28 d are 0.9842 and 0.9874, respectively, nearing 1, thereby indicating high model reliability. The Adjusted R^2^ and Predicted R^2^ for the two models are 0.9638 and 0.8490 and 0.9712 and 0.8657, respectively. The coefficients of variation stand at 3.12% and 2.14%, while the signal-to-noise ratios are recorded at 20.198% and 19.398%, respectively. This confirmation underscores that both models exhibit high accuracy and robust reliability, attesting to the effectiveness of the models established in this study.

**Figure 5:**
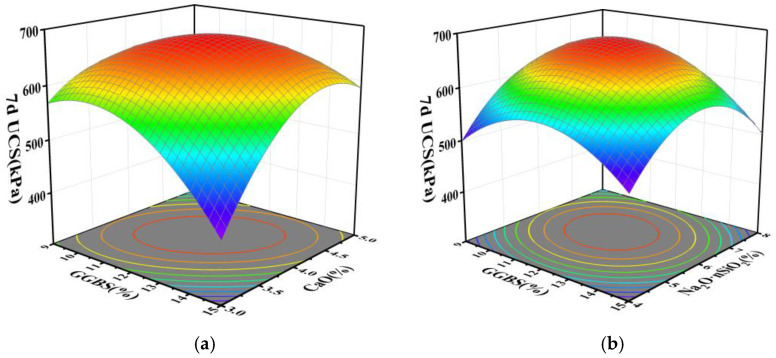

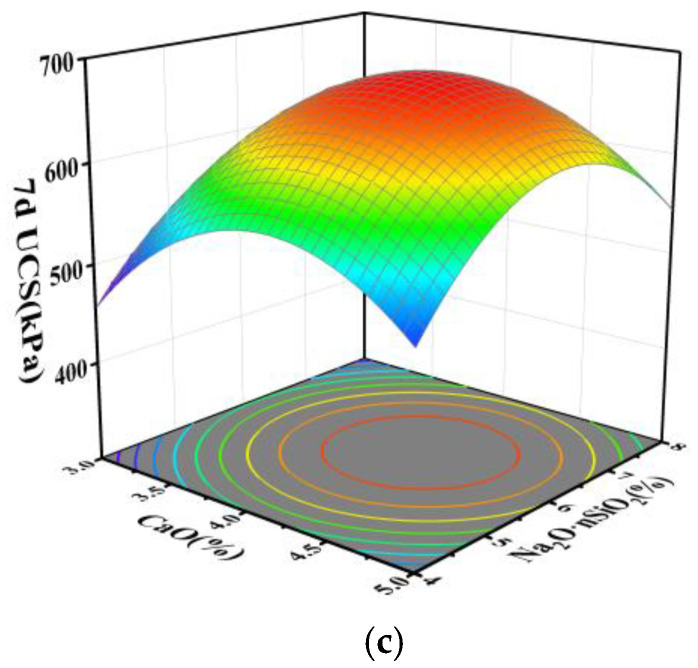
Surface of mutual influence for three factors on 7 d UCS strength: (**a**) AB, (**b**) AC, and (**c**) BC.

**Figure 6:**
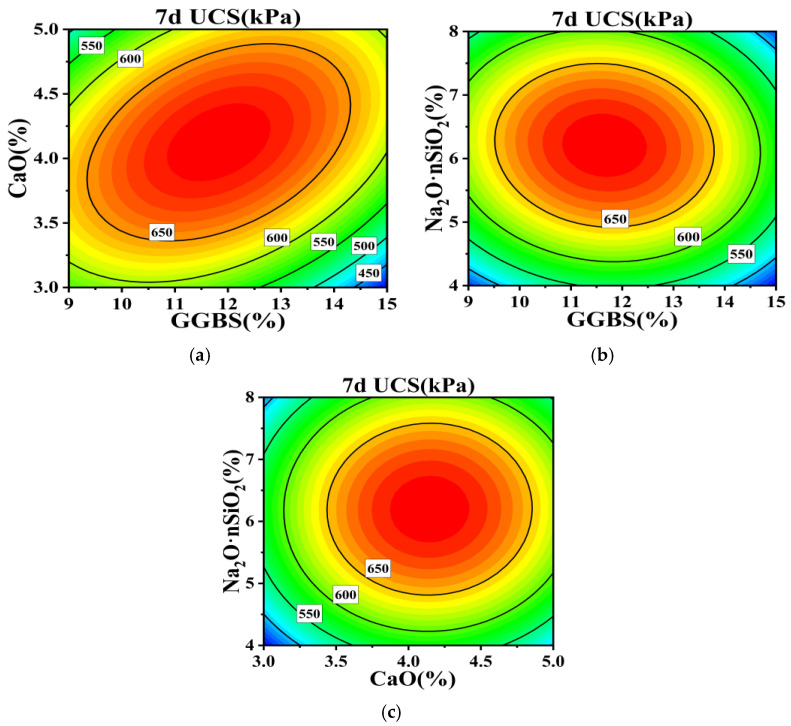
Contour plots of mutual influence for three factors on 7 d UCS strength: (**a**) AB, (**b**) AC, and (**c**) BC.

**Figure 7:**
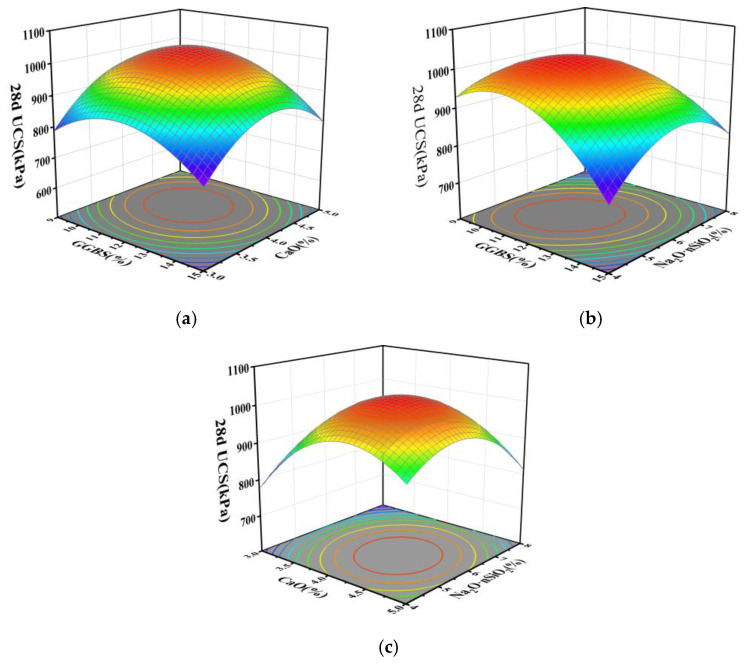
The surface of mutual influence for three factors on 28 d UCS strength: (**a**) AB, (**b**) AC, and (**c**) BC.

**Figure 8:**
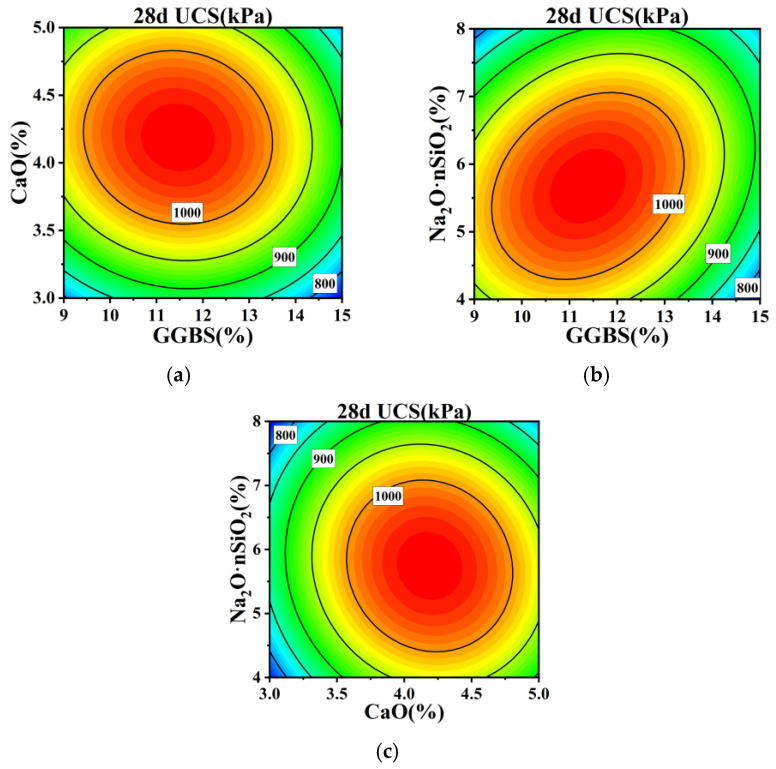
Contour plots of mutual influence for three factors on 28 d UCS strength: (**a**) AB, (**b**) AC, and (**c**) BC.

Employing the regression equation, the configurations of response surface plots and contour maps are scrutinized to analyze the impact of GGBS, CaO, and Na_2_O·nSiO_2_ on unconfined compressive strength. These plots effectively explain the interactions among the variables. By evaluating the steepness of the response surface plots, the magnitude of their impact on response values can be assessed; a steeper gradient signifies more intense interactions among the variables. The interactions of GGBS content (A), CaO content (B), and Na_2_O·nSiO_2_ content (C) with respect to the 7 and 28 d unconfined compressive strength values are delineated through the response surfaces and contour lines depicted in Figure 5, Figure 6, Figure 7 and Figure 8.

Figure 5a–c systematically depicts the trends on interaction surfaces AB, AC, and BC, respectively. On the AB surface, the gradient of the 7 d UCS initially ascends and subsequently descends with increasing GGBS content. At low GGBS levels, this pattern is mirrored with increasing CaO content; however, at elevated GGBS levels, the gradient stabilizes following an initial ascent with increasing CaO content. The optimal 7 d UCS is achieved with GGBS contents between 11–14% and CaO contents between 3.5–4.5%, wherein CaO exerts a more pronounced influence on UCS than GGBS. On the AC surface, the gradient of the 7 d UCS similarly elevates and then diminishes with escalating GGBS and Na_2_O·nSiO_2_ contents, peaking within the ranges of 11–14% GGBS and 5–7% Na_2_O·nSiO_2_, signifying a more substantial influence of Na_2_O·nSiO_2_. On the BC surface, analogous trends manifest with Na_2_O·nSiO_2_ and CaO, where the peak 7 d UCS is noted within CaO contents of 3.5–4.5% and Na_2_O·nSiO_2_ contents of 5–7%, with CaO exerting a more significant effect. Contour plots in Figure 6a–c depict the interaction between GGBS and CaO as notably distinct, forming an elliptical shape, while the interactions involving Na_2_O·nSiO_2_ demonstrate as circular, suggesting less significance and minimal impact on UCS.

Figure 7 and Figure 8 demonstrate that the 28 d cured soil’s compressive strength trend closely mirrors that of the 7 d cured soil. The highest unconfined compressive strength is observed when GGBS, CaO, and Na_2_O·nSiO_2_ contents are within the ranges of 11% to 14%, 4% to 4.5%, and 5% to 7%, respectively. The elliptical contour lines for GGBS and Na_2_O·nSiO_2_ on the contour maps indicate a significant interaction between these components, corroborating the findings from the significance analysis in Table 6.

**Figure 9:**
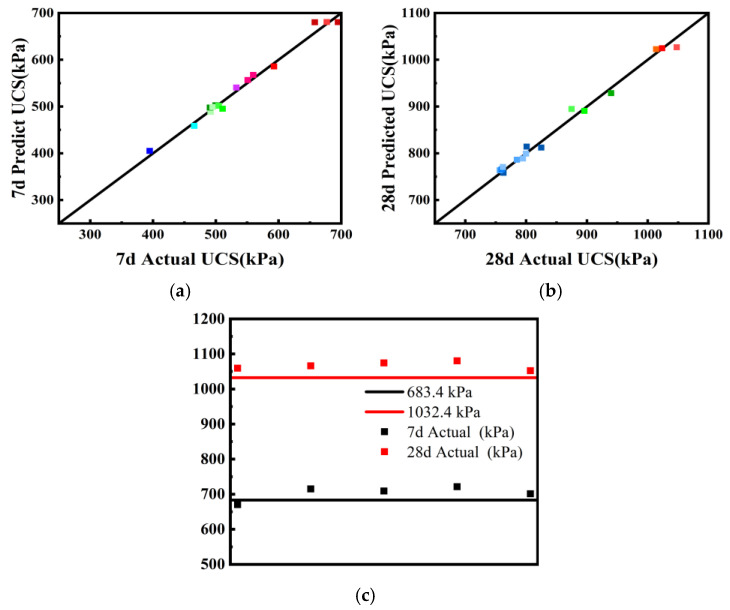
Comparison of predicted compressive strength with actual value; (**a**) 7 d, (**b**) 28 d, and (**c**) optimal ratio.

Design-Expert software was used to optimize the mix proportions of alkali-activated, slag-stabilized dredged silt, targeting the maximum unconfined compressive strength. The optimal mix proportions were established at 11.5% GGBS, 4.1% CaO, and 5.9% Na_2_O·nSiO_2_. Scatter plots comparing the actual versus predicted unconfined compressive strengths for 7 and 28 d, as illustrated in Figure 9a,b, reveal data points closely aligned along a 45-degree diagonal, demonstrating high fidelity in the model’s predictions.

To validate the accuracy of the optimal mix ratio for the cured soil, samples were prepared and subjected to unconfined compressive strength tests under standard curing conditions until the designated ages were reached. The actual and predicted strength values of the cured soil, as detailed in Table 8 and depicted in Figure 9c, indicate that the actual results are derived from averages across five distinct sets of strength tests. The absolute relative error (D) between the predicted and actual strengths, computed using Equation (3), confirms that, for both 7 d (683.4 kPa predicted vs. 703.4 kPa actual) and 28 d (1032.4 kPa predicted vs. 1066.3 kPa actual), the discrepancies are under 5%. This validation underscores the high accuracy of the predictive model developed in this study, offering a reliable reference for subsequent applications.

### 3.3. Analysis of Microstructural Characteristics and Mechanisms

**Figure 10:**
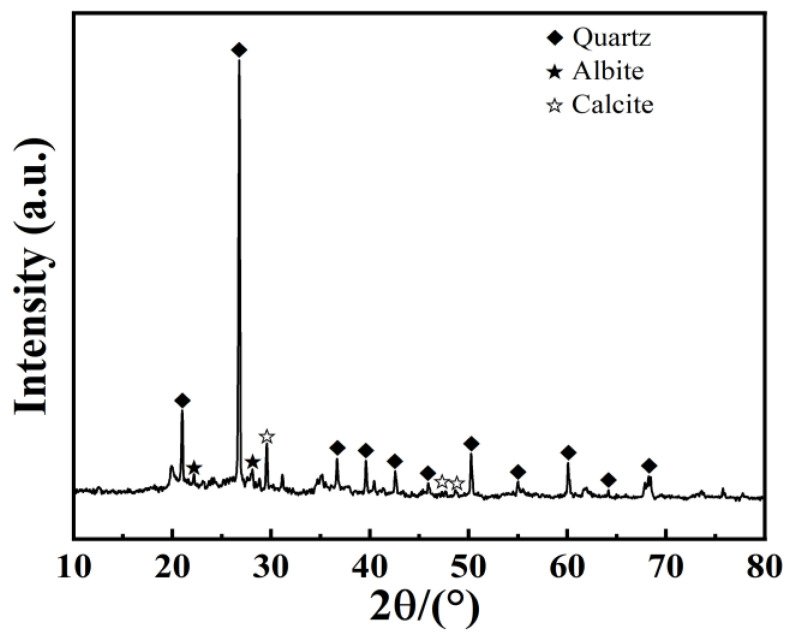
XRD of dredged silt.

X-ray diffraction (XRD) experiments were performed to analyze the alterations in hydration products under diverse curing conditions. The XRD spectrum illustrated in Figure 10 identifies quartz, albite, and calcite as the predominant components of the dredged silt.

**Figure 11:**
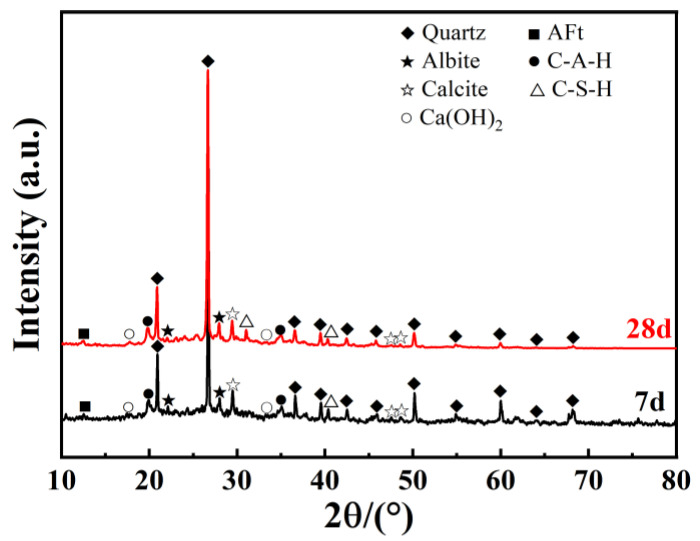
XRD of stabilized dredged silt at 7 and 28 d under the optimal ratio.

The XRD spectrum of the GGBS-CaO-Na_2_O·nSiO_2_-stabilized dredged silt, displayed in Figure 11, indicates an enhanced composition of internal hydration products, extending beyond the foundational constituents of the original dredged silt, including quartz, albite, and calcite. Significantly, new hydration products such as calcium aluminate hydrate (C-A-H), portlandite (Ca(OH)_2_), calcium silicate hydrate (C-S-H), and ettringite (AFt) have been identified. When comparing the 28 d spectrum to the 7 d spectrum, a noticeable decrease in portlandite peaks and an increase in calcite peaks are observed, likely attributable to the consumption of portlandite by pozzolanic reactions and the enhancement of calcite through carbonation as curing progresses. This synergistic interaction within the GGBS-CaO-Na_2_O·nSiO_2_ binder system not only facilitates the formation of new hydration products but also substantially enhances the strength of the stabilized dredged silt. This phenomenon corresponds with the significant factor interactions observed in response surface testing, exemplifying the complex interplay among the components within the binder system.

**Figure 12:**
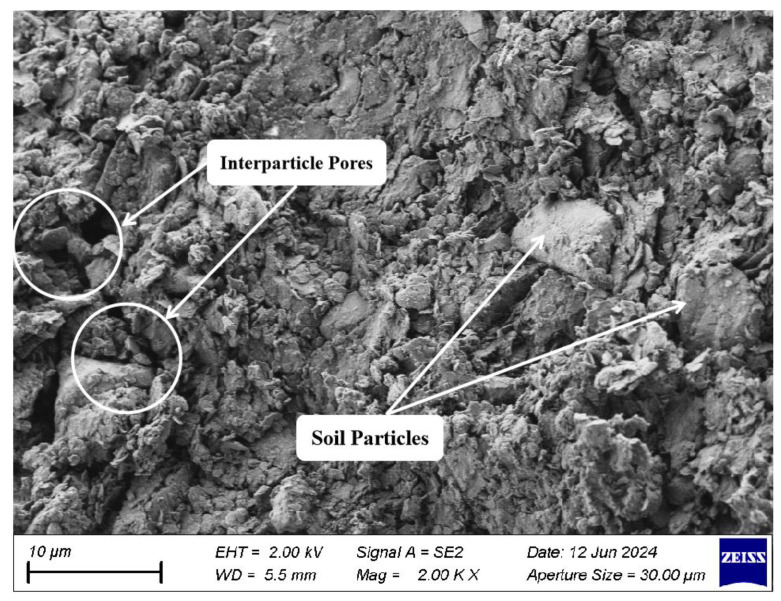
SEM image of dredged silt (2000 times).

Scanning electron microscopy (SEM) was used to investigate the microstructures of both dredged silt and stabilized dredged silt. The SEM image depicted in Figure 12 shows that the particles in the dredged silt are large and poorly interconnected, resulting in a structurally weak composition characterized by abundant pores and cracks. This configuration manifests macroscopically as subpar mechanical performance.

**Figure 13:**
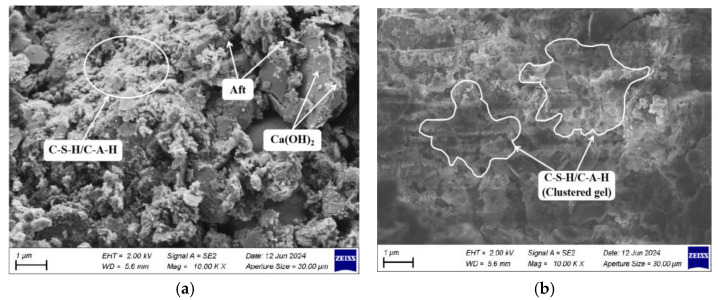
SEM images of stabilized dredged silt under the optimal ratio (10,000 times): (**a**) 7 d and (**b**) 28 d.

Figure 13a,b showcases the SEM images of dredged silt stabilized with an optimal mix of GGBS, CaO, and Na_2_O·nSiO_2_ at 7 and 28 d, respectively. The images explain that, as the curing time progresses, the gaps between soil particles diminish and the structural density escalates, thereby bolstering the overall integrity and compressive strength of the stabilized dredged silt samples. A substantial volume of white flocculent gel-like hydration products is observed adhering between the soil particles. These hydration products proliferate as the curing period extends, culminating in a more compact soil structure. This effect arises from the SiO_2_ and Al_2_O_3_ in both the dredged silt and GGBS gradually dissolving under the influence of the alkaline activators CaO and Na_2_O·nSiO_2_, subsequently reaggregating to yield copious flocculent cementitious materials, namely, C-S-H and C-A-H. These materials adhere to the glassy surfaces, enveloping the soil particles and effectively filling the inter-particle spaces. Additionally, acicular structures (AFt), observed within the pores, function as a framework supporting the soil particles, collaborating with the cementitious hydrates to effectively fill the voids, thereby creating a dense, net-like internal structure that enhances the ongoing improvement in the macroscopic mechanical strength of the stabilized soil. However, the presence of AFt observed in the images is limited, possibly due to the extensive formation of C-S-H and C-A-H, which may envelop or obscure the AFt.

Relative to the SEM image at 7 d, the stabilized dredged silt exhibits substantial changes by 28 d, reflecting the progression of hydration reactions. Na_2_O·nSiO_2_ compounds fully dissolve, enabling the resultant cementitious materials to overlap and progressively form extensive clumped and networked structures. As the boundaries between soil particles diminish, a compact aggregate materializes. This transformation consolidates the microstructure, significantly augmenting the macroscopic mechanical strength of the stabilized dredged silt.

**Figure 14:**
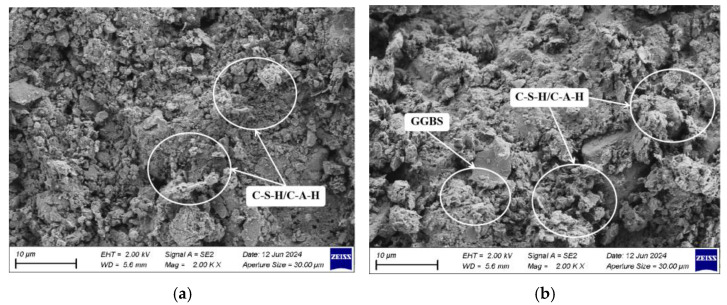
SEM images of stabilized dredged silt with different GGBS contents (2000 times): (**a**) S3 and (**b**) S4.

Further analysis explored the impact of various factors on the microstructure of hydration products using SEM to examine stabilized dredged silt with differing slag contents in the 7 d response surface tests, as depicted in Figure 14. The SEM images from experimental groups S3 and S4, depicted in Figure 14a and Figure 14b, respectively, display differing mix ratios: S3 with 9% GGBS, 5% CaO, and 6% Na_2_O·nSiO_2_ and S4 with 15% GGBS, 5% CaO, and 6% Na_2_O·nSiO_2_. Figure 14b demonstrates a more significant presence of unreacted GGBS compared to Figure 14a. As the GGBS content increases, the micro-porosity between the particles of the stabilized silt diminishes, culminating in a denser soil structure. This densification occurs as Ca-O bonds in GGBS break more readily than Si-O and Al-O bonds, thereby liberating additional Ca^2+^ ions to accelerate the exothermic hydration reactions, hastening the initial dissolution and promoting the formation of supplementary C-S-H and C-A-H.

**Figure 15:**
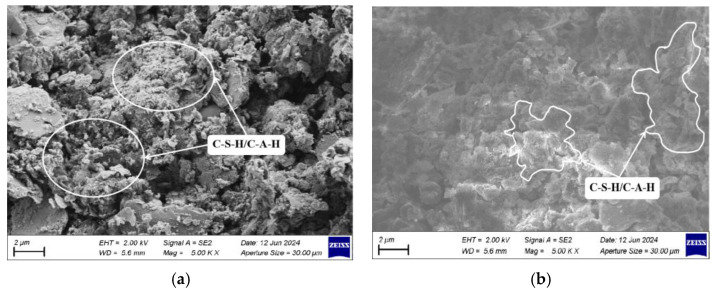
SEM images of stabilized dredged silt with different alkali-activator contents (5000 times): (**a**) S2 and (**b**) S4.

Figure 15a,b presents SEM images for experimental groups S2 and S4, with S2’s mixture comprising 15% GGBS, 3% CaO, and 6% Na_2_O·nSiO_2_. The images clearly demonstrate that an increase in the quantity of alkaline activators significantly reduces the porosity among the particles of stabilized dredged silt and intensifies the density of the white network gel interspersed among the particles. This effect stems from the elevated OH^−^ concentration resulting from increased alkaline activator usage, which accelerates the dissolution of silico-aluminate compounds in both GGBS and dredged silt. This enhancement in hydration reactions leads to the prolific creation of silicate aluminates. Using the adhesive properties of C-S-H and C-A-H, the overall structural integrity of the stabilized silt is substantially enhanced, rendering the strength of the S4 stabilized silt significantly superior to that of S1.

**Figure 16:**
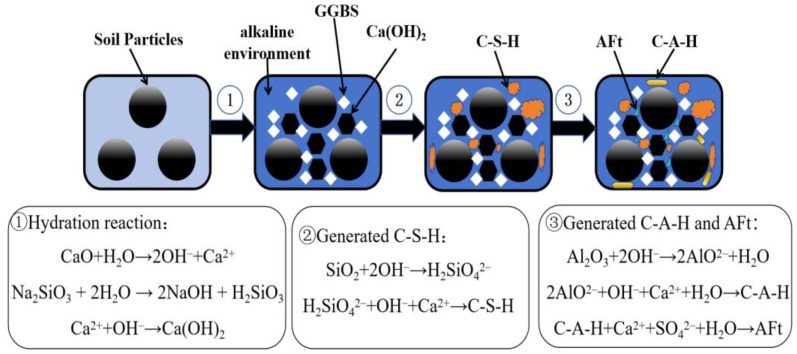
Microscopic mechanism model of stabilized dredged silt.

Based on the extensive XRD and SEM experiments, along with macroscopic testing, a micro-mechanism model for the GGBS-CaO-Na_2_O·nSiO_2_ stabilization of dredged silt has been developed, as illustrated in Figure 16. The stabilization involves several key processes:

Process ①: When the GGBS-CaO-Na_2_O·nSiO_2_ solidifier is incorporated into the dredged silt and thoroughly mixed, the CaO within the solidifier undergoes rapid hydration, releasing large amounts of Ca^2+^ and OH^−^. Concurrently, Na_2_O·nSiO_2_ reacts with water to produce abundant OH^−^ and Na^+^. As the concentration of these ions rises, Na^+^ and K^+^ from the silt particles dissolve and engage in adsorption exchange with Ca^2+^, leading to a reduction in the double-layer thickness of the soil particles and decreasing their separation. This process results in flocculation and the formation of larger aggregates, enhancing the soil particles’ cohesion. Meanwhile, the less soluble flake-like product, Ca(OH)_2_, gradually precipitates, further enhancing the bonds between soil particles due to its hydration activity combined with the filling effects of GGBS particles. The marked increase in OH^−^ levels in the solution thus establishes an advantageous alkaline environment conducive to facilitating processes ② and ③.

Process ②: Under alkaline conditions, SiO_2_ in the dredged silt initially reacts with OH^−^ ions to form H_2_SiO_4_^2−^. This compound then further reacts with OH^−^ and Ca^2+^ to generate the flocculent gel C-S-H. The C-S-H gel envelops and binds the soil particles, leading to the formation of larger particle aggregates.

Process ③: Under alkaline conditions, the activity of GGBS is enhanced, causing Al_2_O_3_ contained within it to undergo a hydration reaction with OH^−^ ions in the solution, forming AlO^2−^. This ion then combines with Ca^2+^ to create the flocculent gel C-A-H, which serves as a binder for the soil particles. Additionally, C-A-H reacts with SO_4_^2−^ present in the dredged silt, leading to the formation of needle-like structures known as AFt.

Overall, the stabilization of dredged silt via GGBS-CaO-Na_2_O·nSiO_2_ is propelled by the binding properties of C-S-H and C-A-H, coupled with the filling actions of Ca(OH)_2_ and AFt. These components synergistically transform the loose soil into a dense aggregate, significantly enhancing its stability and mechanical strength.

## 4. Conclusions

To facilitate the resource utilization of dredged silt and industrial waste, it is stabilized using blast furnace slag, activated calcium oxide, and sodium silicate. The stabilization and micro-mechanisms of GGBS-CaO-Na_2_O·nSiO_2_ composite materials were investigated through single-addition tests, response surface methodology, X-ray diffraction, and scanning electron microscopy experiments. The main conclusions are as follows:

(1) In the single-addition experiments, CaO showed the most significant stabilization effect, characterized by rapid hydration reactions and early strength development; the stabilization effect of GGBS was substantially lower than that of CaO, with a gradual increase in strength over the curing period; Na_2_O·nSiO_2_ only slightly improved the strength of the stabilized soil at dosages below 6%, with most strength gains occurring in the later stages of curing.
(2) Based on response surface methodology, regression fitting and significance analysis of the experimental results yielded regression equations for the 7 d and 28 d compressive strengths of the stabilized silt, revealing significant interactions between GGBS-CaO and GGBS-Na_2_O·nSiO_2_. Optimization of the regression equations determined the optimal mix proportions for the GGBS-CaO-Na_2_O·nSiO_2_ interaction affecting the stabilized silt to be 11.5% GGBS, 4.1% CaO, and 5.9% Na_2_O·nSiO_2_.
(3) The hydration reactions within the GGBS-CaO-Na_2_O·nSiO_2_ composite binder system are mutually enhancing, producing hydration products such as Ca(OH)_2_, C-S-H, C-A-H, and AFt in an alkaline environment. These products encapsulate, bind, and fill, stabilizing the microstructure of the soil.

## Figures and Tables

**Table 1:** Basic physical properties and indexes of dredged silt.

Moisture Content/%	Relative Density	Liquid Limit/%	Plastic Limit/%	Plasticity Index	Liquidity Index
59.2	2.61	46.1	25.9	20.2	1.64

% is a mass fraction.

**Table 2:** Main chemical components of the experimental materials.

Materials	Mass Fraction/%				
	CaO	SiO_2_	Al_2_O_3_	SO_3_	Fe_2_O_3_
Dredged silt	3.2	62.6	19.5	1.3	6.5
GGBS	42.0	33.0	12.0	1.67	1.0

**Table 3:** Single-addition test scheme.

Stabilizing Agents	Additive Percentage/%	Curing Period (Days)
GGBS	3, 6, 9, 12, 15	7, 14, 28
CaO	2, 3, 4, 5, 6	
Na_2_O·nSiO_2_	2, 4, 6, 8, 10	

**Table 4:** Response surface design scheme.

Independent Variable	Code Level		
	−1	0	1
A (GGBS)/%	9.00	12.00	15.00
B (CaO)/%	3.00	4.00	5.00
C (Na_2_O·nSiO_2_)/%	4.00	6.00	8.00

**Table 5:** Regression model coefficient and significance.

Sample	Independent Variable			Response Value	
	A/%	B/%	C/%	Y_7d_/kPa	Y_28d_/kPa
1	−1	−1	0	561	785
2	1	−1	0	395	763
3	−1	1	0	511	875
4	1	1	0	593	800
5	−1	0	−1	495	940
6	1	0	−1	492	757
7	−1	0	1	551	795
8	1	0	1	505	801
9	0	−1	−1	466	762
10	0	1	−1	491	896
11	0	−1	1	500	760
12	0	1	1	533	825
13	0	0	0	695	1014
14	0	0	0	658	1013
15	0	0	0	677	1024
16	0	0	0	695	1013
17	0	0	0	677	1048

**Table 6:** Regression model coefficient and significance.

Source of Variance	Y_7d_			Y_28d_		
	Coefficient	F-Value	*p*-Value	Coefficient	F-Value	*p*-Value
Model	680.4	48.31	<0.0001	1022.4	61.02	<0.0001
A	−16.62	7.29	0.0306	−34.25	26.8	0.0013
B	25.75	17.49	0.0041	40.75	37.94	0.0005
C	18.13	8.67	0.0216	−21.75	10.81	0.0133
AB	62.00	50.7	0.0002	−13.25	2.01	0.1997
AC	−10.75	1.52	0.2568	47.25	25.5	0.0015
BC	2.00	0.053	0.8249	−17.25	3.4	0.1078
A^2^	−76.08	80.35	<0.0001	−102.08	125.28	<0.0001
B^2^	−89.33	110.77	<0.0001	−114.58	157.85	<0.0001
C^2^	−93.57	121.57	<0.0001	−97.07	113.31	<0.0001
R^2^	0.9842			0.9874		

**Table 7:** Model reliability test analysis.

Group	Std.Dev /kPa	Mean/kPa	R^2^	Adjusted R^2^	Predicted R^2^	C.V./%	Adequate Precision
7 d	17.41	558.53	0.9842	0.9638	0.8490	3.12	20.198
28 d	18.71	874.76	0.9874	0.9712	0.8657	2.14	19.398

**Table 8:** Model reliability test analysis.

A/%	B/%	C/%	28 d			7 d		
			Yp/kPa	Ya/kPa	D/%	Yp/kPa	Ya/kPa	D/%
11.5	4.1	5.9	683.4	703.4	2.92	1032.4	1066.3	3.30
$D=\frac{\left|Ya-Yp\right|}{Ya}\times 100\%$								(4)

Note: Ya: actual value, Yp: predicted value.

## Data Availability

All data generated or analyzed during this study are included in this published article.
